# Challenges Facing Home-Based Caregivers in the Management of Health Care Risk Waste

**DOI:** 10.3390/ijerph15122700

**Published:** 2018-11-30

**Authors:** Thobile Zikhathile, Harrison Atagana

**Affiliations:** 1 Mangosuthu University of Technology, Faculty of Natural Sciences, 511 Griffiths Mxenge Highway, Umlazi, KwaZulu-Natal 4031, South Africa; 2 Institute for Science and Technology Education, University of South Africa, Pretoria 0003, South Africa; atagahi@unisa.ac.za

**Keywords:** health care risk waste, home-based caregivers

## Abstract

The quadruple burden of diseases, early discharge from hospital and hospital at home have resulted in home-based care services becoming a requirement in South Africa. These home-based care services generate a significant amount of health care risk waste that is mismanaged. More attention is given to the health care risk waste generated in hospitals and clinics than to health care risk waste generated by home-based caregivers. Therefore, this study investigates the health care risk waste management practices by home-based caregivers. The study adopted a mixed research approach, qualitative and quantitative methods, using a literature review, interviews, and questionnaires as means of data collection. Results show that there are different types of health care risk waste generated as a result of different activities performed by home-based caregivers, but that the waste was found to be managed in an unsafe manner. The majority of households receiving home-based care did not have basic sanitation facilities such as toilets, running water and waste removal services, aggravating the issue of health care risk waste mismanagement. The study recommends a new policy framework that will lead to safe management practices of generated health care risk waste to be adopted by home-based caregivers.

## 1. Introduction

South Africa is currently experiencing what is termed by the National Department of Health a quadruple burden of diseases that are chronic in nature [1]. Quadruple burden of disease means that South Africa is experiencing a mixture of four colliding epidemics. These epidemics are human immunodeficiency virus/acquired immunodeficiency syndrome (HIV/AIDS) and tuberculosis, maternal and child mortality, non-communicable diseases, and violence leading to injuries and trauma [1]. HIV/AIDS have severely affected South Africans [2]. The burden of diseases, especially HIV/AIDS, places stress on the already strained health care facilities [1]. There are numerous people that require medical attention as a result of this quadruple burden, and their number is increasing on a daily basis [3]. Both the public and the private health care facilities are strained as a result [4]. However, the public health care facilities are more strained than the private health care facilities in terms of financial, human and equipment resources [4]. This is because the majority of South Africans rely solely on public health care facilities because they are living below the poverty line and are therefore unable to afford private health care [5]. 

The strain has resulted in appalling conditions in public health care facilities, which are in dire need of upgrading [5]. To ease the burden on health care facilities, the South African government reintroduced home-based care services [6]. Home-based care is an old concept that dates back to 1813; however, it is now gaining more prominence and becoming widespread [7]. Home-based care is a primary health programme and is recognised as the most cost-effective strategy for delivering essential health care programmes to communities [8]. Services that are offered are nursing care, physical care, patient support, domestic chores, and psychological care. Nursing care includes dressing wounds, administering medication, and supervised treatment support (directly observed treatment support). Physical care includes assisting patients to use toilets, feeding and bathing patients, changing patients, and helping patients exercise. Domestic chores include collecting water for patients, and cleaning and laundry for patients. The psychological care includes health education, counseling, rehabilitation, and praying. Support includes referring patients to health care facilities, identifying people who do not have any source of income, and bereavement counseling [9]. It also provides promotive and preventative services [8]. The services of home-based care are directed at all people who may be in need of care, such as frail older people, people with moderate to severe functional disabilities, people recovering from illnesses who are in need of assistance, terminally ill persons, persons living with HIV/AIDS or any other chronic diseases and any other disadvantaged group [7].

People that work in the home-based care programme are referred to as home-based caregivers [10]. They are generally physically fit middle-aged women from the community [11]. The home-based caregivers do not have medical training but undergo home-based care training [12]. The training introduces them to various aspects of home-based care such as diseases and disability, management of patients’ condition and treatment, assisting patients with mobility and preventing complications, and patient referral [12]. The training offered is focused on nursing care, life skills, counseling, case-finding, and record-keeping [13]. It is designed to capacitate and equip caregivers to provide necessary help for the community they serve [14]. 

The services provided by home-based caregivers yield numerous benefits [15]. However, home-based caregivers face the challenge caused by the generation of health care risk waste [15]. There are large volumes of health care risk waste arising from care given by home-based caregivers and currently there are no arrangements made to correctly manage the generated waste [16]. The management of health care risk generated by home-based caregivers is a mounting problem, especially in developing countries such as South Africa that have inadequate resources and lack of cost-effective waste disposal processes [16]. 

Health care risk waste is defined as the waste that is generated during the process of providing health care services to patients [17]. The waste is considered to be very hazardous and is ranked the second most hazardous after radioactive waste because it has the potential to cause adverse health effects and significant pollution in the environment [18]. In South Africa, the waste generated by home-based caregivers is managed in the same manner as general domestic waste [19]. The waste is either discarded in an open field, burnt, buried in shallow graves or collected by or on behalf of the local authority to be disposed of in solid waste land fill sites [19].

Health care risk waste management in a safe and efficient manner is important and it is the responsibility of all generators, including home-based care givers. This is for the protection of the environment, preservation of human health, and to comply with all waste management laws such as the National Environmental Management: Waste Act. However in South Africa, Kenya, and Botswana health care risk generated by caregivers is managed at the discretion of caregivers and the management practices are usually unsafe [20,21,22]. Mismanagement of heath care risk waste is a common occurrence in the developing countries, far different from the developed countries [22]. The situation is worse with the health care risk waste generated in homes. The South African government has focused monitoring and control of health care risk waste generated in health care facilities [23], but pays no attention to the health care risk waste generated in homes [16]. The situation is completely different in the developed countries. According to the Royal College of Nursing, in the United Kingdom (UK) health care staff working in the community are responsible for managing the health care risk waste that they generate and are required to fully comply with duty to care. In Japan and UK, health care risk waste generated by care givers is either left in a safe and secure place in the house where it was generated to be collected by designated contractures; or collected and transported in an approved container by caregivers to health care facilities [22,24]. 

There have been very few studies on health care risk waste generated by home-based care givers. This paper therefore seeks to investigate health care risk waste management practices by home-based care givers in their daily operations. It seeks to investigate by first evaluating home-based care activities that generate health care risk waste and thereafter quantifying the generated health care risk waste. As this study is focused on the health care risk waste generated by home-based care givers, the intention is to bring attention to this this overlooked situation. The anticipation is the altering of the current procedure and cause formulation of new policies of health care risk waste management by home-based care givers.

## 2. Materials and Methods

Research methods of collecting data for this research study had two approaches, that is, using primary data sources and secondary data. Research methods for this research using primary data include two instruments: questionnaires and interviews. Semi-structured interviews was designed for the home-based care manager. The secondary data was obtained from books, journal articles, government publications, published material, and the internet. The research design for this research required the use of the listed tools. In the first qualitative phase of the study, the objective, was to understand the home-based care phenomenon, the knowledge of care givers on health care risk waste, identify the activities carried out by care givers that lead to health care risk waste generation, and the attitude of care givers toward health care risk waste issues. The topics discussed answered these questions.

In the second quantitative phase of the research, using questionnaires, data was collected from the sampled home-based care givers employed by the Department of Health and working at Umlazi Township. Questionnaires were administered in July 2016. The first section was on the demographics of the home-based care givers. The second section focused on conditions of employment and workload. The third was on their knowledge of health care risk waste. The fourth was on the different types and quantities of health care risk waste generated. The fifth was on management practices of the generated health care risk waste. The questionnaires were for identifying the type of health risk waste generated by the care givers, and for analysing the management practices of the generated waste. 

Questionnaires were translated to the local language, IsiZulu. The questionnaires had closed format questions and contained five sections: demographics of caregivers and their patients; home-based care services and workload; protective clothing, different types and quantities of generated waste; management practices of the generated waste; and types of training received. 

The person interviewed was prepared prior to the interview for the interview process. The questions were open-ended, and the respondent was required to answer in her own words. The open-ended questions allowed the interview process to be flexible and made the interviewee comfortable. The responses were recorded in writing because in general people are not comfortable with being taped while they are talking. 

There are two managers of home-based care givers at Umlazi, and only one manager was available during the time of collecting data. The interviews were conducted to obtain data on the background of the home-based care givers to have an elaborate understanding of the work of home-based care givers. With the understanding of their work, the researcher was able to grasp how the waste was generated. The interviews were conducted to get an understanding of the support received by the care givers from their employer, the Department of Health. This support is very important because it includes training of care givers on health care risk waste, among other things. The interviews were done to get an understanding on the reporting structures of home-based care and monitoring and evaluation. Monitoring and evaluation would ensure that the care givers carry out their duties efficiently and are accountable for any wrongdoing such as mismanagement of the waste. 

The questions that were used in the interview sessions were about home-based care givers and the patients that were receiving nursing care from them. These questions were similar to the questionnaires. However, they required more elaborate responses. They were specifically focused on the work of the home-based care givers. They were also on health care risk waste generation and management practices. Since the interviews required elaborate answers, the research also enquired about the working conditions and the challenges faced by the home-based care givers and how the challenges impact on their work. The secondary data was obtained from books, journal articles, government publications, published material and the internet.

### 2.1. Data Validity and Reliability

Face and content validity were established before questionnaire distribution. The questionnaires were submitted to an expert in the field, an Environmental Health Practitioner, whose area of operation is Umlazi Township. She has over twenty years’ experience in the field of environmental health (home-based care and health care risk waste). The purpose of submitting the questionnaires was for an assessment of the content validity of the questionnaire. The questionnaire was also submitted to the Research Ethics Committee of the Mangosuthu University of Technology, and the committee made a few suggestions that were intended to include language translation. With the assistance of an isiZulu expert, at the Mangosuthu University of Technology, the questionnaire was translated to isiZulu, the Umlazi Township local language.

The language used was very simple, and the questionnaire had very short questions to avoid confusing respondents. It can, therefore, be concluded that the tool used was reliable. Content validation helped to get the draft questionnaire moderated so that the questionnaire was reliable.

For reliability of survey instruments, the questionnaire was tested to avoid ambiguity and anything that might have confused the respondents when answering the questions. The instrument used for testing the internal consistency of responses was Cronbach’s alpha score. The reliability test was implemented within SPSS statistical package (2.3, Armonk, NY-USA: IBM Corp), and the outcomes of the reliability analysis are presented in the final chapter.

The research instrument was tested using a pilot study before the questionnaires were distributed to the research participants. The questionnaires were submitted to eight home-based care givers who were not participating in the research. The home-based care givers were randomly selected, and all worked in Umlazi Township. The pretest results reflected questions that were not clear or not understood. Minor changes were found to be necessary (2016/CAES/015).

### 2.2. Ethical Consideration

The rights and identities of the study participants were protected. They did not include their identities on the questionnaires. The participants participated in the study voluntarily, and their information was kept anonymous and confidential. The study adhered to the ethical guidelines of the University of South Africa and the Department of Health. A research ethical clearance application was submitted to the University of South Africa, and the Department of Health. Ethical clearance was granted by the College of Agriculture and Environmental Science Research Ethics Review Committee of the University of South Africa for the research to be done (2016/CAES/015).

### 2.3. Permission

Before applying for ethical clearance from the University of South Africa, the researcher had to obtain permission from the KwaZulu-Natal Department of Health to conduct the study. This is because the research subjects, home-based care givers, were employees of the KwaZulu-Natal Department of Health. Application for permission was requested in writing. The letter explained the research, the purpose and the objectives of the study. The KwaZulu-Natal Department of Health granted permission for the study.

### 2.4. Consent

Consent for this research was obtained. A consent letter was attached to the questionnaires. It was written in English and translated to IsiZulu because the respondents are isiZulu speakers. The purpose of the consent letters was for thoroughly debriefing the participants of the study. The consent letter introduced the research and research topic. It further explained the purpose and benefits of the research. The participants were asked to participate in the research, and it was explained how they were chosen to be in the study. The letter further explained that their participation was strictly voluntary and they could withdraw if they wanted to. The consent letters were for the questionnaires and the interview respondents.

### 2.5. Study Setting

The study area was Umlazi, a South African township established in 1845 to accommodate black labourers [25]. Umlazi Township is on the east coast of the province of KwaZulu-Natal, which shares borders with three Southern African Development Community (SADC) countries; Lesotho, Mozambique and Swaziland. Umlazi has approximately one million residents [26] and both formal and informal settlements. It is experiencing rapid growth in the number of informal settlements as a result of people migrating to the greater Durban area for employment opportunities [26]. 

Umlazi is not sufficiently provided with basic infrastructure and services such as health care facilities, sanitation facilities, safe drinking water, waste collection services and electricity [27,28]. The imbalances of the past as a result of the apartheid system saw areas such as Umlazi completely neglected in terms of service delivery [27]. Decades after democracy, delivery of basic services still remains a huge challenge. 

Another great challenge in Umlazi is the prevalence of HIV/AIDS, tuberculosis (TB), a high rate of unemployment, poverty, violence and crime [29]. However, the greatest challenge is the HIV/AIDS epidemic [1], which makes the situation in Umlazi dire [10]. The epidemic has resulted in the deaths of economically active people, leaving many orphans and elderly people to look after themselves [10]. There is only one hospital in the south of Durban, which caters for the entire population of the south of Durban, the estimated one million Umlazi residents [25], and the other residents from the south of Durban. The high rate of unemployment and poverty limits the residents from accessing private health care facilities. The residents therefore rely heavily on the public health care system. However, the one hospital cannot accommodate all people that require medical assistance. This means the residents to rely on the services of home-based caregivers [30].

### 2.6. Participants

The study only targeted home-based care givers employed by the Department of Health and not the entire population of Umlazi. The reasons for selecting home-based care givers employed by the department of health were the following: Department of Health care givers are formally registered caregivers, and the non-government care givers are formed and dissolved constantly making it difficult to collect data from them. There were 219 home-based care givers and two managers at the time of data collection. Therefore, for questionnaires the study population was 219 home-based caregivers employed by the Department of Health in Umlazi Township. The questionnaire survey instrument targeted home-based care givers employed by the Department of Health because the study was targeting employees employed in a formal manner and respondents that would be available for the entire duration of the research.

The study population for interviews was managers of home-based care givers who are also professional nurses. There were two managers of home-based care givers in Umlazi Township employed by the Department of Health. One manager was selected to participate in the study. Purposive sampling, explained by Kumar (p. 207) as deliberately selecting the cases that will be studied, was applied [31]. The interview respondent was purposefully selected because the other manager was unavailable to participate in the research for health reasons. The questionnaire survey instrument targeted home-based care givers employed by the Department of Health because the study was targeting employees employed in a formal manner and respondents that would be available for the entire duration of the research. The Statistical Package for the Social Sciences (SPSS) method was used to calculate the sample size population for this research.

### 2.7. Data Collection Procedure

Oral interviews were used to obtain data from the available home-based care manager. The selected interviewee was interviewed four times over a period of six months, from January to July 2016 at Maweleni office in Umlazi. The duration of each session was approximately 30 min for each section. The questions were open-ended and required lengthy narratives from the interviewee. The questions allowed for flexibility in response and were also probing in nature as is encouraged for qualitative research. This is because probing deepens the response to questions and increases the quality of responses. 

The questionnaires were used to obtain survey data from the caregivers. A database of total population of registered caregivers was extracted from the home-based care giver population, registered in the department of Health, into MS Excel spreadsheet. Thereafter, a representative sample of caregivers was selected by random probability sampling using the random sampling functionality in MS. Excel. Caregivers in the sample were administered survey questionnaires with the assistance of the manager of home-based caregivers at Umlazi.

### 2.8. Data Analysis

The respondents were given questionnaires. The questions in the questionnaire were numerically coded. Using the numerical codes, the responses from the respondents were entered into a spreadsheet. The data entered on the spreadsheet was then checked for accuracy and transferred into SPSS. The data was then analysed to provide outputs such as frequency tables, histograms, and charts.

### 2.9. Data Validity and Reliability

Face and content validity were established before questionnaire distribution by submitting them to an Environmental Health Practitioner (EHP) who works with caregivers, whose area of operation is Umlazi and Isipingo Townships. She has over twenty years of experience in the field of Environmental Health (home-based care and health care risk waste). She took the questionnaires to her colleagues. The purpose of submitting the questionnaires to an EHP was to conduct a pilot survey so as to assess the content validity of the questionnaire. EHPs read the questionnaire and made suggestions about it. The questionnaire was also submitted to the Research Ethics Committee of the Mangosuthu University of Technology, which made a few suggestions that were intended to include language translation. With the assistance of an isiZulu expert, the questionnaire was translated to isiZulu, the Umlazi Township local language.

The language used was very simple, and the questionnaire had very short questions so as to avoid confusing respondents. It can be concluded from the pilot study that the tool used was reliable as the content validation helped to make the draft questionnaire so.

The instrument used for testing the internal consistency of responses was Cronbach’s alpha score. The reliability test was implemented within the SPSS statistical package. The research instrument was tested using a pilot study before the questionnaire was distributed to the research participants by submitting it to eight home-based caregivers who were not participating in the research. These home-based caregivers were randomly selected, and all worked in Umlazi Township. The pretest results reflected questions that were not clear or not understood. This meant minor changes were necessary to those questions in the pilot study.

## 3. Results

The sample size was 80, and all 80 questionnaires were returned answered. Interviews were conducted with one home-based care manager.

### 3.1. Demographics of Caregivers

The majority of caregivers were middle-aged females. From the study sample only two caregivers were males while 78 were females. Majority (37) of caregivers are between the ages of 41 and 50 years old, followed by 24 caregivers between 31 and 40 years old. The last group of 16 are between 51 and 60 years old. There are no caregivers below the age of 31. Mean age is 43. Only 32 caregivers had completed Grade 12. All other caregivers dropped out of high school. None of the caregivers had tertiary qualifications. The majority of the caregivers had vast experience as caregivers. Only six had less than five years of experience.

#### 3.1.1. Gender

Out of the eighty (80) participants that were selected for the study, seventy-seven (78) were women and two (2) were men.

#### 3.1.2. Age

Results from the study show that the age of the care givers ranged between twenty (20) and sixty (60). The majority of the home-base care givers between the age group of thirty-one (31) and fifty (50). Mean age of care givers is 43, derived from the calculation below:

(1) $$\mathrm{Mean}=\frac{\Sigma \mathrm{fx}}{\Sigma f}$$

Σfx = sum of frequency and age(2)

Σf = sum of frequency(3)

(4) $$\frac{\Sigma \mathrm{fx}}{\Sigma f}=\frac{3473.50}{80}$$

Means = 43.41875

#### 3.1.3. Education Level

The majority of care givers who participated in the study did not complete grade 12 (12), only thirty-two (32) had completed grade twelve (12). None of the care givers who participated in the study had university qualifications.

#### 3.1.4. Work Experience

Responses to the question on the recruitment of care givers indicates that the Department of Health recruits community members to work as care givers, because community members are familiar with the area, have the same cultural affiliation and speak the same language as the patients and their relatives. It was also preferred that the care givers stay within a walking distance from the homes of the patients.

The majority of the care givers had between six (6) and ten (10) years’ work experience of home-based care. Some had worked as care givers for over twenty (20) years. Only six had worked as care giver for less than six (6) years, thirty-six (36) had worked for between six (6) and ten (10) years, twenty-four (24) for between eleven (11) and fifteen (15) years, ten (10) for between sixteen (16) and twenty (20) years, and two (2) for between twenty-one (21) and twenty-five (25) years.

### 3.2. Demographics of Patients

Care givers have eighty (80) patients each a month, however in the month of July one care giver had 86 patients. These demographics are based on the eighty patients receiving services from the eighty (80) participants. The home-based care givers indicated that the majority of their patients were female. The female population of the care givers was 4772 compared to the male population which was 1634 and the majority of their patients were between the age of 40 and 60 years. Some of their patients had never been to school. Of the patients who attended school, only 2500 patients completed grade 12, and only 600 had a tertiary education qualification. The majority of their patients were unemployed (5200). Patients lived on government grants (2700) and pensions (3100), others had no means of income.

### 3.3. Services Provided by Home-Based Caregivers

According to the manager, home-based caregivers provide different types of services depending on the needs of the patient. The services are nursing care, physical care, patient support, psychological care, and domestic chores for their patients. The services that generate health care risk waste are nursing care and physical care. On average the study participants generated 2.2 kg of health care risk waste individually and 183.75 kg of health care risk waste combined on the day of data collection.

### 3.4. Workload of Home-Based Caregivers

An interview response on the number of houses a day that home-based care givers is not fixed. It is based on the duties they need to perform and the needs of the patients that they are servicing. The home-based care givers were therefore asked to answer questions related to their work based on the day they were completing the questionnaire. The response on the question on workload of care givers showed that care givers visit on average visited three (3) to four (4) houses on the day of data collection with each house having two to three chronically ill patients.

An interview question was also asked on how care givers get to know about patients that require their services. The response was that the care givers work on a referral system. The health care facilities has a list of people that require the assistance of care givers and they then contact the managers of care givers and forward contact details and the address of the patient that needs the services. The managers then contact care givers that work in the area to make a follow up and the patients will be included on the patients’ database. Sometimes it is community members who will identify people that need the services of care givers. The community members then inform the care givers in the area about the patient or household.

#### 3.4.1. House Visit

The majority of the care givers on average visited a few houses on the day of data collection. Thirty-nine (39) and thirty-one (31) care givers visited three and four houses respectively. Four (4) visited up to five houses a day.

#### 3.4.2. Number of Patients Attended to Per Household

Fifty-one (51) care givers attended to one patient per household. However, some care givers attended to more than one patient per household. Four (4) care givers attended up to three patients per household nursing care.

### 3.5. Protective Measures Used during Caregiving

**Figure 1:**
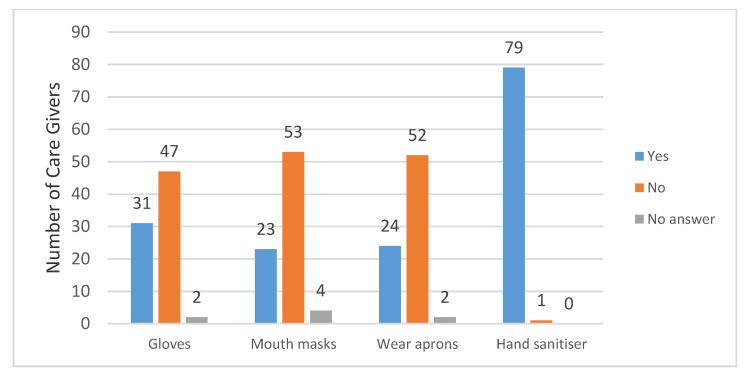
Use of protective gear by caregivers.

Most of the caregivers do not use any protective gear when working. Data obtained showed that 47 of the caregivers do not wear gloves, 53 do not wear mouth masks, and 52 do not wear aprons. However, 79 of them use hand sanitisers after nursing patients. The hand sanitiser is used as a form of protection to prevent contamination (Figure 1).

### 3.6. Generation of Health Care Risk Waste

The interview responses showed that care givers generated health care risk waste every time they provided nursing and physical care to patients and that different types and quantities of health care risk waste were generated.

The different types of health care risk waste generated in a day include used aprons, gloves, cotton, gauze, plasters, nappies, needles, syringes, toilet paper, medical containers, expired medication, human waste, vomit, sputum, pus, soiled linen, and plastic bags. Home-based care givers do not inject patients, however they assist the patients to self-inject. Therefore needles wastes are not generated by home-based care givers but they are responsible for the management after they have assisted patients to self-inject.

### 3.7. Quantities of Health Care Risk Waste Generated by Home-Based Care Givers

Caregivers were asked to fill generated health care risk on the day of data collection in a plastic bag. The plastic bags were approximately 32 × 50 cm in size. Each plastic bag given by care givers was weighed on a scale to quantify the health care risk waste generated by each home-based care giver on the day of data collection. The weight was rounded off to the nearest number to avoid decimals.

Thirteen (13) caregivers collected 0.5 kg, thirty one (31) collected 1 kg, twenty five 25 collected 2 kg, nine (9) collected 3 kg, and two (2) collected 4 kg (Table 1). Total amount of health care risk waste that was collected by home-based care givers on the day of data collection was 183.75 kg. Therefore the average health care risk waste generated by home-based care givers is 2.3 kg a day.

### 3.8. Management of Health Care Risk Waste

The interview response on health care risk waste management was that the generated waste that was left in the homestead was included with domestic waste. It was stored in domestic waste containers or plastic bags for a week. The houses are not equipped with waste storage facilities or areas therefore waste is stored outside the homes. The waste is then collected by Durban Solid Waste to be disposed of in domestic waste landfill site. 

The interview response on the health care risk waste management practices, the handling, storage, and disposal practices by care givers was that the care givers use different methods to dispose of the generated health care risk waste. The care givers chose the most suitable methods for disposing of the health care risk waste.

**Figure 2:**
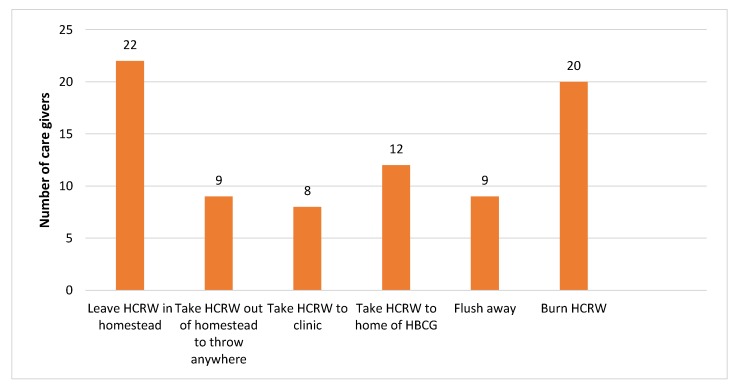
Methods of disposal of health care risk waste used by caregivers.

Questionnaire data collected indicates, as shown in Figure 2, that 22 caregivers leave the generated waste at the homestead where it was generated, nine throw the waste anywhere deemed fit by them, eight take the waste to the nearest health clinics to be disposed along with other health care waste, 20 burn the waste, and nine flush or discard it in a pit latrine. The health care risk waste left in the household is either stored in plastic bags or general waste containers for approximately one week.

### 3.9. Training

**Figure 3:**
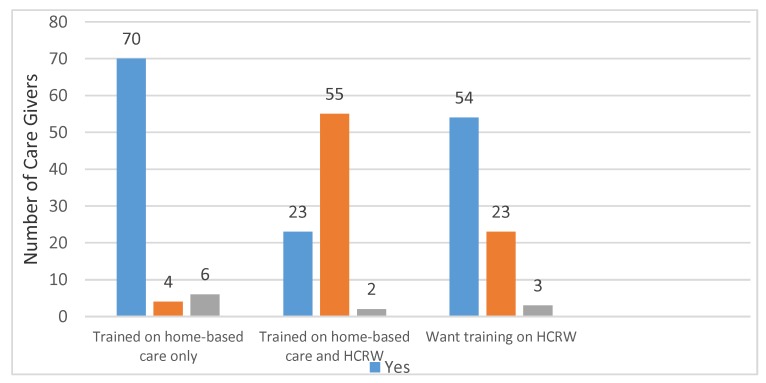
Training.

According to the caregivers, training has not been provided for everyone. Only 70 caregivers had received training on home-based care before they assumed their duties; and four caregivers indicated that they did not receive any form of training (Figure 3). Furthermore, 55 caregivers said they have never been trained on health care risk waste management, while 23 reported that they had been trained (Figure 3); 54 of them want to be trained on health care risk waste management.

## 4. Discussion

### 4.1. Demographics

The findings revealed that home-based care is mostly offered by middle-aged women. This is because the services offered are physically demanding and not suitable for the elderly to administer. About 60% of the home-based caregivers had completed their basic education level. They are therefore literate and can be educated on home-based care and health risk waste matters.

### 4.2. Services

Services provided by caregivers, as stated above, are nursing care, physical care, patient support, domestic chores, and psychological care. Nursing care includes dressing wounds, administering medication, and supervised treatment support (directly observed treatment support). Physical care includes assisting patients to use toilets, feeding and bathing patients, changing patients, and helping patients exercise. Domestic chores include collecting water for patients, and cleaning and washing for patients. The psychological care includes health education, counseling, rehabilitation, and praying. Support includes referring patients to health care facilities, identifying people who do not have any source of income, and bereavement counseling. The services that mostly generate health care risk waste are nursing care and physical care. As physical care includes bathing patients, and assisting them to go to the toilet, the water used may be contaminated. The contamination may be either body secretions or blood resulting in the used water being health care risk waste. The materials used during nursing care such as bandages, cotton wool and gauze are often contaminated with human secretions such as pus and blood. Nappies used by bedridden patients, and cotton wool and bandages used for treating wounds contain human secretions and are health care risk waste. Discarded medication containers are also health care risk waste. 

Results from the study further revealed that home-based caregivers provide counseling to patients and members of the family with sick people. However, counseling does not generate health care risk. People with terminal illnesses needed to be counseled so that they can cope with the disease. It is also done to assist family members to cope with living with a terminally ill person and with a death in the family. 

Furthermore, home-based caregivers conducted health education that is very broad and incorporates numerous health issues. The most common health issues, though, were HIV/AIDS and TB, and education was given both on prevention and protection. The approach of the South African government to HIV/AIDS is prevention through health education, therefore education is the key vehicle used to curb the scourge. The home-based caregivers were also used to conduct health education on the importance of the use of drugs such as antiretroviral therapy and TB drugs on HIV/AIDS in general. They were also used to conduct education on mother to child HIV/AIDS transmission, and breast feeding.

Home-based caregivers are used to reduce the patient medication defaulter rate through health education by ensuring that patients take their medication as required by the Department of Health. There is a very high medication defaulter rate, especially among TB patients [1] and home-based caregivers play a significant role in ensuring through health education that the patient adheres to the treatment plan. Assisting patients to take their medication generates health care risk waste because the containers where medication is kept are health care risk waste. Health education is also conducted to prevent and control other communicable diseases. 

The home-based caregivers support communities tremendously by identifying families and patients that do not have any source of income. They mobilise donors to donate food to those families and refer patients to health care facilities that offer the required care. 

Home-based caregivers are used extensively by the Department of Health to trace TB cases, to communicate with the public, to relay vaccination programmes, etc. The home-based caregivers literally work as ears and eyes for the Department of Health. They also do a lot of referrals, where they refer community members who required treatment to the appropriate health care facilities.

### 4.3. Workload

Based on the results, on average caregivers visit between three and four houses a day. Each house visited has on average two patients. The majority of the patients are illiterate and very poor. Patients prefer to be nursed by caregivers because they are often humiliated and abused by nurses in clinics and hospitals. The abuse by nurses occurs because socio-economic differences between nurses and patients are huge [32]. Furthermore, public healthcare facilities are in an appalling state. 

The results indicate that services offered generate health care risk waste. Therefore, the increased workload increases the amount of health care risk waste. Health care risk waste generated is a mixture of items used for nursing patients and contaminated protective clothing. This generated healthcare risk waste is mostly unaccounted for.

### 4.4. Protective Measures Used during Caregiving

Protective measures are items such as specialised clothing that is used to protect both home-based caregivers and the patients they are treating. The protective clothing prevents cross-contamination by micro-organisms and communicable diseases. It is the protective clothing that becomes contaminated during the treatment of patients instead of the health care personnel. All health care personnel must wear protective clothing when treating patients because they have to be protected from anything that endangers or causes harm to their health [27]. The home-based caregivers who answered the questionnaires indicated that they do not all wear protective clothing, such as the gloves, mouth masks, and aprons listed in the *Health and Safety Act (1993)* [33], during caregiving. This absence of working protective clothing is common in developing countries [32]. The caregivers only used hand sanitisers to clean their hands after nursing a patient. This leaves both the patients and home-based caregivers exposed to infections. The inadequate resources for health care workers, fraud and corruption, a weak economy, and low management capabilities, are the main reasons for home-based caregivers not receiving appropriate protective clothing. 

There were, however, home-based caregivers (almost 40%) who indicated that they had protective clothing at the time data was collected, but the provision thereof was not reliable, it was done randomly. Hand sanitiser is used by almost 100% of home-based caregivers because they buy the sanitiser themselves. Any disposable protective clothing used is regarded as infectious and becomes health care risk waste because it is often contaminated with blood and or other body fluids [33]. The disposable medical protective clothing, such as gloves, aprons and face masks, has to be discarded after use. Hand sanitisers are antimicrobial and therefore can contaminate the environment. The containers that keep the sanitisers will have residues of the sanitiser and the containers are therefore health care risk waste [34].

### 4.5. Generation of Health Care Risk Waste

The amount and composition of the health care risk waste generated by caregivers is dependent upon the number of patients nursed and the services provided. The caregivers indicated that on some days they generate aprons and gloves, but not every day because they sometimes did not have them to wear when providing nursing care to patients. They sometimes have to improvise by wearing plastic bags for protection. Another form of medical waste is a syringe, although caregivers indicated they are not authorised by the Department of Health to administer injections. The syringes are used by diabetic patients and caregivers only assist these patients when they administer the injections themselves. They do, however, assist patients to take medication orally and in the process may use syringes for accurate measurements. The syringes are recycled, meaning they are used several times, before they are discarded and are therefore not generated daily as health care risk waste. The medical containers and expired medication are not generated every day either. Some caregivers indicated that they generate soiled linen and plastic bags, which they use as protective clothing. The generated health care risk waste is typically contaminated with fecal matter and body fluids such as urine, blood, vomit, pus, sputum and phlegm. There is also waste water generated from personal and household care of the patients and also from cleaning containers used to contain sputum, phlegm and vomit from patients with nausea and TB. The different types of generated health care risk waste were not weighed because the caregivers weighed all health care risk waste together daily.

The quantity of generated health care risk waste indicated by caregivers is not enormous, about 1.5 kg a day (see Table 1). However, these indicated quantities can be debated because caregivers fail to record the health care risk waste that they generate properly, resulting in a great challenge in obtaining accurate data. Another issue is inadequate training on health care risk waste resulting in caregivers failing to properly define and classify the waste and therefore failing to quantify the waste properly and so the generated waste ends up being included with domestic waste.

This has resulted in limited information on health care risk waste generated by home-based caregivers, which causes the precise estimate of the waste to be unknown. However, desk top studies indicate that it is quite substantial and increasing sharply [35,36,37,38]. This is a result of the current trend of home health, hospital at home, and early discharge of patients [39]. The hospital at home, and early discharge of patients is caused by lack of human and financial resources to accommodate all the patients who require medical attention. Data from the study could therefore not be compared with any other available data.

Health care risk waste, regardless of the quantity, has a potential to cause severe damage to the environment and the health of individuals [40,41]. Furthermore, the general practice of caregivers was to include the generated health care risk waste with domestic waste. This practice has serious environmental and health implications as the health care risk waste contaminates the domestic waste resulting in all the waste becoming hazardous [42].

### 4.6. Management of Health Care Risk Waste

**Figure 4:**
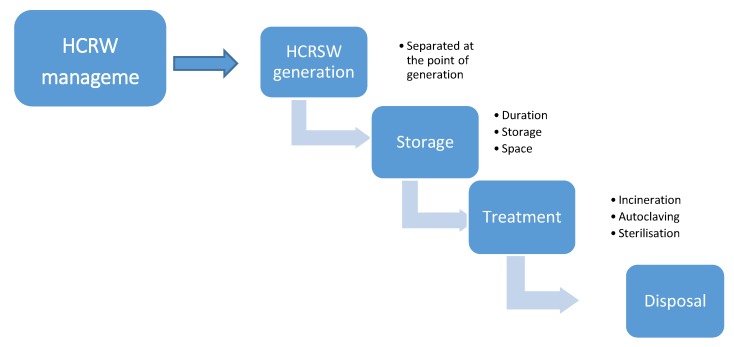
Process of health care risk waste.

Only 10% of caregivers managed health care risk waste in a proper manner. They took the generated waste to the nearest healthcare facility to be stored, collected, treated and disposed of safely. The majority of the home-based caregivers managed the generated waste in an unsafe manner that can potentially endanger health and pollute the environment. Some caregivers discarded the waste in open spaces, some burnt the waste, some flushed or discarded it in a pit latrine, and some included the waste with domestic waste. However, all generated health care risk waste should follow a specific process after generation, the waste management process, the term management referring to the storage, treatment and disposal of the generated health care risk waste [43]. The process of health care risk waste management is shown in Figure 4. Management of waste is defined as the processes involved in dealing with the waste of humans and organisms, including minimisation, handling, processing, storage, recycling, transport and final disposal [34].

#### 4.6.1. Separation

**Figure 5:**
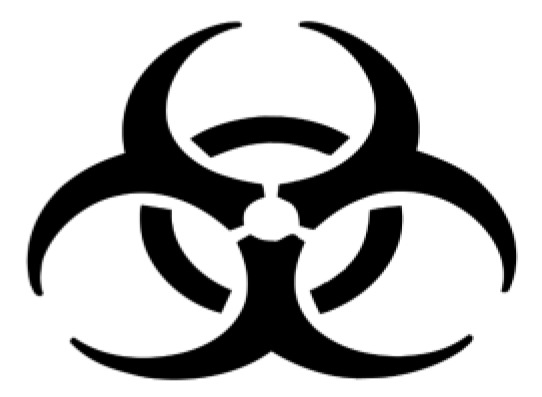
International infectious substance symbol as adapted. [18].

Over 90% of caregivers do not separate the generated health care risk waste at source but include the waste with domestic waste. However, all generated health care risk waste should be separated at the point of generation and placed in correct colour-coded containers with the international infectious substance symbol (Figure 5) to facilitate safe handling and treatment according to the level of hazard [44].

#### 4.6.2. Storage

As the caregivers did not separate the generated waste at source, it was included with the domestic waste and stored in either domestic waste containers or plastic bags. The containers used by caregivers are those supplied by Durban Solid Waste for general waste and are not designed to store health care risk waste. Also, the plastic bags referred to are not the colour-coded plastic bags recommended by the South African National Standards (SANS) 10248 [45], but those created to store general waste. Therefore, the containers used by home-based caregivers place the lives of waste workers in danger of needle pricks and other related injuries. It further exposes the workers and the general public to different infections, such as hepatitis and TB. It also exposes the environment to contaminants. The health care risk waste stored in home contravenes the guidelines by the KwaZulu-Natal Department of Health [43], which are impossible to meet because homes are not designed to accommodate health care risk waste [19,21].

The majority of health care risk waste generated by caregivers does not have proper storage facilities and is stored for the same period as the general waste generated by the household. In formal settlements the duration of storage of waste is a week as it is included with the general waste, and Durban Solid Waste collect the general household waste once a week.

#### 4.6.3. Collection

Waste collection differs considerably between formal and informal households. For the formal settlements, waste is collected by or on behalf of Durban Solid Waste. 

For the informal households, the residents do not have appropriate means of disposing of either domestic waste or human waste. Waste collection is irregular and random. The residents are often without options when it comes to waste disposal. Some residents take generated waste to a common waste receptor. Sometimes weeks go by without the garbage being collected. Waste in these receptors often overflows and scatters on the ground. The wind blows the waste or it is dragged by domestic animals and spread all around the community. Some residents resort to illegal practices of waste disposal such as disposing waste along the roadside, nearest forest, bush or vacant plot, while others bury it in shallow pits or burn it in their backyards. The shared sanitation facilities are sometimes vandalised, and are usually distant from the homes. The human waste such as urine, sputum, and vomit is discarded in open yards that are accessible to the public, children and domestic animals. 

Health care risk human waste is discarded in toilets and transported by the municipal sewer system to the waste water treatment plant by Durban Solid Waste vehicles. These vehicles are designed to transport domestic waste and not health care risk waste. The drivers are not trained to handle and transport health care risk waste and are not equipped to deal with emergency situations such as preventing or controlling spills or leakages.

#### 4.6.4. Treatment

The only health care risk waste that is treated is the waste that is taken by the 10% of caregivers to the nearest health care facility. It enters the health care risk waste stream to be collected by Compass Waste, as all the health care risk waste emanating from the Department of Health care facilities in Umlazi Township is collected by Compass Waste to be treated before disposal. Different methods, such as incineration, autoclaving, chemical disinfection, and microwave irradiation are used by Compass Waste to treat the health care risk waste.

#### 4.6.5. Disposal

Most of the generated health care risk waste is disposed of in an unsafe manner, either being discarded in open dumps, incinerated or collected by Durban Solid Waste.

### 4.7. Contributing Factors to Health Care Risk Waste Mismanagement

The factors contributing to health care risk waste mismanagement range from ignorance, lack of capacity and resources, attitude, cultural beliefs, lack of support, and irregular waste collection by waste collectors. The most significant contributing factor is ignorance because caregivers do not receive adequate training and do not have adequate knowledge of health care risk waste management. Some of the health care risk waste knowledge by caregivers is self-taught and such training is usually not accurate. Caregivers are not provided with training specific to health care risk waste. Some of the training received by caregivers incorporated aspects of health care risk waste but were not specific to health care risk waste.

### 4.8. Risks of Health Care Risk Waste Mismanagement

Mismanagement of health care risk waste has a direct and indirect impact on health and the environment. The indirect health impact generally occurs from needle stick injuries and transmission of infectious agents [41]. These impacts generally occurs as a result of waste management activities such as collection, handling, transportation, treatment, or disposal of waste [46]. The indirect impact on health results from the environmental contamination, land and water, caused by untreated health care risk waste. Health care risk waste contaminated environment cause diseases such as dysentery, typhoid, bilharzia, malaria, cholera, and parasite worms. Indiscriminately disposed health care risk waste provides breeding grown for disease cause vectors that spread diseases.

## 5. Recommendations

Recommendations for efficient and effective health care risk waste management practices by home-based care givers. The study offers a list of recommendations made to improve the working procedures and conditions of care givers. A health care risk waste management plan after an intense consultative process with all relevant stakeholders is required. The recommended plan needs to be detailed, explaining how the generated health care risk waste will be classified and separated at source from the domestic waste. It first needs to describe the activities of care givers that generate health care risk waste. Thereafter, explain how the generated health care risk waste will be quantified and effectively managed, including handling, storing, and disposal. It has to specify the roles and responsibilities of the all the stakeholders in the proper management of health care risk waste and accountability and punitive measures to be taken against mismanagement of health care risk waste.

### 5.1. Monitoring of the Generated Health Care Risk Waste

Results from the research indicate that the generated health care risk waste by home-based care givers enters the general waste stream. The main reasons for this practice are traceable to ignorance and unavailability of resources to handle the generated health care risk waste adequately. The work of care givers needs to be monitored closely to identify activities that generate health care risk waste. This can assist with the monitoring of waste produced by care givers. The monitoring will help with resource allocation and will further support the generation of prevention and protection measures of the public and the environment.

### 5.2. Resource Allocation

Management of health care risk waste by home-based care givers needs to be allocated financial and human resources. Suitably qualified people should be employed so that they can ensure that waste generated by care givers is managed effectively. The type of storing of health care risk waste separately from domestic waste suggested by the 2005 World Health Organisation guidelines require appropriate resources [47].

### 5.3. Development of Policies and Guidelines

Significant health and environmental catastrophes may be experienced if current health care risk waste management continue. The irreversible damage caused by improper management of health care risk waste has already been done especially to the environment. It can, however, be stopped from getting worse. The policies should stipulate the correct procedures for health care risk waste management so that everyone may have common and standard health care risk waste management procedures. They should also include the manner in which management practices will be monitored and evaluated and invoke consequences for failure to comply.

### 5.4. Government and Departmental Collaboration and Cooperation

Government departments should work in a cooperative and transparent manner with one another in order to have a good working relationship primarily on issues of health care risk waste. The government departments must work together to promote the health of care givers, waste workers, and the community by ensuring that they do not get injured by the generated health care risk waste. They should also prevent environmental degradation and pollution by ensuring that the environment is protected from the generated health care risk waste. All departments within the health sector are essential in health care risk waste management. However, the agencies that may play a critical role are the environmental health practitioners, waste management experts, and infection control practitioners. These departments have insights into the current challenges resulting from the mismanagement of health care risk waste. They can also assist with community education, and offer adequate support for the care givers in proper health care risk waste management.

### 5.5. Training of Home-Based Care Givers

Training on health care risk waste should be offered to all care givers, patients and the community at large, however such training should be mandatory. Different platforms such as public meetings and gatherings can be used to educate the public. Over and above being trained, the community must always be reminded of health care risk waste management issues. The following media can be used to remind the public: aggressive marketing on billboards and transport systems, radio and television advertisements, handing out pamphlets and fliers, doing campaigns, etc. 

Training will ensure that both the care givers and the community have an in-depth understanding of the implication of health care risk waste mismanagement on the health of the people and the environment.

### 5.6. Community Education

Ongoing education of the community on health care risk waste management and the impact of mismanagement health care risk waste on human health and the environment is important. Aspects of health care risk waste must not be the responsibility of the care givers only, but the patients receiving nursing services from care givers should take the same amount of ownership, if not more. The community as a whole should also take full responsibility because they will also be affected by the mismanagement of health care risk waste.

### 5.7. Assessment and Auditing

Assessments and auditing of health care risk waste generated by home-based care givers should be carried out to carry it out regularly. Assessment and auditing would be important for the verification of the quantities of health care risk waste generated and disposal of the generated health care risk waste by the care givers. The reports from assessments and auditing will enable the management to assess their capacity to manage the health care risk waste appropriately. This would prevent the current situation of health care risk waste generated by care givers being unaccounted for.

### 5.8. Recommendations for Further Research

There has been very little research conducted on health care risk waste in developing countries, and the available research is inadequate. In South Africa there is a dearth of information regarding home-generated health care risk waste, especially health care risk waste generated as a result of home-based care activities. There is therefore a need for further research on home generated health care risk waste. This may result in proper planning and resource allocation for health care risk waste management so as to protect the health of individuals and the environment. 

There is also a need for further research on the impact that health care risk waste generated by home-based care givers has on waste employees that collect and dispose of domestic waste, waste handlers that recycle the waste, and waste scavengers. There is also a need for further research on the accurate impact that health care risk waste generated in homes has had on the health of people and animals that may be exposed to the waste and the environment.

## 6. Conclusions

The study found that the current South African Health Care System, especially because of the quadruple burden of diseases, are inadequate to manage the health needs of the people. And yet the services provided by the home-based caregivers will continue to be a necessity in the near future. As mentioned, caregivers play a significant role in improving the lives of people and promoting their health. It is only appropriate that their working procedures be re-evaluated and changed so that they do not harm the environment and people’s health. As has been revealed by this study, a considerable amount of health care risk waste generated by caregivers is managed in a manner that endangers the environment and the lives of all those exposed to the waste. Findings of this study show that the health care risk waste management of home-based caregivers is not practiced in accordance with best practices as approved by the Department of Health.

The offered recommendations by the study are to improve the working procedures and conditions of caregivers to protect health, preserve the environment, and comply with the law. Suggested recommendations include: having a health care risk waste management plan, monitoring the generated health care risk waste, resource allocation, development of policies and guidelines, government and departmental collaboration and cooperation, community education, training of caregivers on health care risk waste, and continuous assessment and auditing. However, the most important suggestion is the need for further research that will shed light on the areas of focus and the best way for dealing with the management of health care risk waste.

## Figures and Tables

**Table 1:** Quantity of health care risk waste generated by caregivers per day.

Number of Caregivers	Number of Bags Per Day
13	0.5
31	1
25	2
9	3
2	4

Note: One full bag weighs approximately 1.5 kg.

## Supplementary Materials

Supplementary File 1
