# Supplementary material for: Challenges Facing Home-Based Caregivers in the Management of Health Care Risk Waste
Source: Int J Environ Res Public Health. 2018 Nov 30;15(12):2700. doi: 10.3390/ijerph15122700 (PMC6313601; doi:10.3390/ijerph15122700)
Supplement: Supplementary File 1 [file ijerph-15-02700-s001.pdf]

QUESTIONNAIRE FOR THE HOME-BASED CARE GIVER

Thank you for participating in this research. Please cross the most suitable answer. Example: ☒ X

Gender of HBCG: Male ☐ Female ☐

Age group: 20-30 ☐ 31-40 ☐ 41-50 ☐ 51-60 ☐ 61-70 ☐

Education level (grade): 8 ☐ 9 ☐ 10 ☐ 11 ☐ 12 ☐

Tertiary education: Certificate ☐ Diploma ☐ National Diploma ☐ Degree ☐

Name of the qualification: ....

WORK

Number of years employed: 0-5 ☐ 6-10 ☐ 11-15 ☐ 16-20 ☐ 21-25 ☐ 26-30 ☐ 31-35 ☐ 36-40 ☐

Employed full time: Yes ☐ No ☐

WORK LOAD

Houses you visit a day: 1 ☐ 2 ☐ 3 ☐ 4 ☐ 5 ☐ 6 ☐ 7 ☐

Highest number of patients in a house: 1 ☐ 2 ☐ 3 ☐ 4 ☐ 5 ☐ 6 ☐ 7 ☐

PATIENTS

Gender of patients: Numbers of females ☐ Numbers of males ☐

Education level of patients: No schooling ☐ Schooling ☐ Tertiary ☐

Employment of patients: Number of employed ☐ Number of unemployed ☐

Means of income for the patients: Salary ☐ Pension ☐ Business ☐

Government grant ☐ No income ☐

HEALTH CARE RISK WASTE

What is the health care risk waste that you generate in a day?

Health care risk waste	Yes	No	Sometimes
Gloves			
Aprons			
Cotton wool			
Bandages			
Gauze			
Plasters			

Nappies			
Pads			
Needles			
Syringes			
Toilet paper			
Medication containers			
Expired medication			
Other			

List other: ____

Amount of health risk waste you generate in a day

Half a bag ☐ 1 full bag ☐ 2 full bags ☐ 3 full bags ☐ 4 full bags ☐ 5 full bags ☐

What do you do with the generated health care risk waste?

Leave it in the homestead ☐ Flush the waste ☐ Burn the waste ☐

Throw it away ☐ Take it with you to the clinic ☐ Take it home with you ☐

Storage containers of waste

Plastic bags ☐ Waste storage containers ☐ Both ☐

Health care risk waste training

Have you been trained on health care risk waste? Yes ☐ No ☐

Do you want training on health care risk waste? Yes ☐ No ☐

PROTECTIVE CLOTHING

Do you wear gloves when you are working? Yes ☐ No ☐

Do you wear mouth masks when you are working? Yes ☐ No ☐

Do you wear aprons when you are working? Yes ☐ No ☐

Do you sanitise your hands after each visit? Yes ☐ No ☐

Have you been injured when you are working? Yes ☐ No ☐

WORKING CONDITIONS

Are you provided counseling? Yes ☐ No ☐

If yes, are the counseling sessions sufficient?

Yes ☐ No ☐

THANK YOU
